# Efficacy and Safety of Antithrombotic Therapy With Oral Anticoagulants in Real-World Elderly Patients With Acute Coronary Syndrome and Atrial Fibrillation

**DOI:** 10.3389/fcvm.2022.923684

**Published:** 2022-06-29

**Authors:** Yangxun Wu, Haiping Liu, Liu'an Qin, Yuyan Wang, Shizhao Zhang, Ziqian Wang, Yuting Zou, Tong Yin

**Affiliations:** ^1^Institute of Geriatrics, The Second Medical Center and National Clinical Research Center for Geriatric Diseases, Chinese PLA General Hospital, Beijing, China; ^2^Medical School of Chinese PLA, Chinese PLA General Hospital, Beijing, China

**Keywords:** acute coronary syndrome, atrial fibrillation, oral anticoagulant, elderly, antithrombotic treatment

## Abstract

**Background:**

The efficacy and safety of antithrombotic treatment with oral anticoagulants (OACs) in elderly patients with comorbidities of acute coronary syndrome (ACS) and atrial fibrillation (AF) are unclear.

**Methods:**

A cohort of hospitalized elderly patients (≥65 years of age) diagnosed with ACS and AF and treated with oral antithrombotic agents were consecutively recruited. Follow-up was performed for at least 1 year. Major adverse cardiac events (MACEs) were defined as a composite of all-cause death, nonfatal myocardial infarction (MI), nonfatal stroke, and systemic embolism. The safety outcomes of bleeding were defined according to the Bleeding Academic Research Consortium (BARC) criteria.

**Results:**

A cohort of 548 eligible patients (76 ± 6.6 years) was analyzed. Compared to the patients with OAC treatment (*n* = 184, 33.6%), patients treated without OAC (*n* = 364, 66.4%) were older, had a lower prevalence of persistent AF and unstable angina (UA), and more often presented with paroxysmal AF, acute myocardial infarction (AMI), stent implantation and dual antiplatelet therapy (DAPT). Compared to the patients without OAC treatment (*n* = 364, 66.4%), patients treated with OAC (*n* = 184, 33.6%) had a lower risk of MACEs at both the 1-year (4.3 vs. 15.1%, adjusted HR: 0.34, 95% CI: 0.15–0.80, *p* = 0.014) and 5-year (17.5 vs. 48.4%, adjusted HR: 0.36, 95% CI: 0.19–0.67, *p* = 0.001) follow-up. No significant difference was observed for bleeding events of BARC ≥2 between the groups (8.0 vs. 9.0%, adjusted HR: 1.17, 95% CI: 0.58–2.34, *p* = 0.667). Compared with warfarin-treated patients, the non-vitamin K antagonist oral anticoagulant-treated patients had lower risks of all-cause mortality (2.1 vs. 9.5%, HR: 0.18, 95% CI: 0.03–0.98, *p* = 0.047) and bleeding events of BARC ≥ 3 (2.1 vs. 4.8%, HR: 0.14, 95% CI: 0.02–1.10, *p* = 0.062).

**Conclusions:**

Antithrombotic therapy with OACs in elderly patients with ACS and AF was associated with a lower risk of ischemic events without an increase in bleeding events. In real-world practice, the clinical awareness of anticoagulation treatments in elderly patients with ACS and AF needs to be strengthened.

## Introduction

The comorbidity of acute coronary syndrome (ACS) and atrial fibrillation (AF), which occurs mostly in elderly patients (1–5), contributes to the increased risk of all-cause death and stroke (1, 2, 6–8). Antithrombosis with dual platelet inhibition or oral anticoagulation (OAC) is most frequently recommended for the prevention of ischemic stroke and mortality in patients with ACS and AF (9). In the recent guideline from the European Society of Cardiology (ESC), long-term administration of OACs, including non-vitamin K antagonist oral anticoagulant (NOAC) or vitamin K antagonist (VKA), is recommended for medically treated or percutaneous coronary intervention (PCI)-treated patients with ACS and AF (9). When considering the use and duration of combined antithrombotic therapy in patients with ACS and AF, the concomitant risks of ischemic events and antithrombotic treatment-related bleeding need to be carefully balanced (10), especially in elderly patients with an increased risk of both bleeding and ischemic events (11, 12). Of note, as only 7% of patients in ACS trials were reported to be elderly (≥75 years) (13), evidence-based recommendations for antithrombotic therapy in elderly patients with ACS and AF have rarely been reported.

Although there is sufficient evidence supporting the use of OACs in elderly patients with AF (14–16), poor adherence to guideline-directed anticoagulation has been reported (17). In addition, physicians are less likely to prescribe OACs in this population due to the risk of bleeding (18–21). OAC treatment in real-world elderly patients with ACS and AF has rarely been reported. Observational studies found that compared to antithrombotic therapy in ACS patients without AF, antithrombotic therapy in patients with ACS and AF was less appropriate and led to more adverse outcomes (22, 23). The situation might be worse in elderly individuals, who are more likely to have multiorgan damage, increased ischemic and bleeding risks, a high incidence of comorbidities and comedication, and decreased adherence to prescriptions (1, 3, 24). Therefore, the present study aimed to investigate the efficacy and safety of antithrombotic therapy with OACs in real-world elderly patients with ACS and AF.

## Methods

### Patients

Patients aged ≥65 years old with a diagnosis of the comorbidities of coronary artery disease (CAD) and AF were recruited consecutively from the Department of Cardiology, Chinese PLA General Hospital, from 2010 through 2017. Participants were eligible for inclusion if they had both ACS and nonvalvular AF and were treated with oral antithrombotic agents, including antiplatelet agents and/or anticoagulants. ACS, including unstable angina (UA), non-ST elevation myocardial infarction (NSTEMI), and ST-elevation myocardial infarction (STEMI), were defined according to the 2018-ESC criteria (10). Nonvalvular AF refers to AF without a mechanical artificial heart valve or with moderate to severe mitral stenosis (usually derived from rheumatism) (9). Nonvalvular AF could be paroxysmal or persistent but not secondary to a reversible disease. Participants were excluded if they had reversible causes of AF, known contraindications to the use of antithrombotic regimens or a life expectancy of no more than 6 months. Patients with any indication for OAC other than AF (e.g., mechanical heart valves, pulmonary embolism, and left ventricular mural thrombus) or who were lost to follow-up were also excluded. This study was performed in accordance with the Declaration of Helsinki and was approved by the Ethics Committee of Chinese PLA General Hospital, and all patients provided written informed consent. Eligible patients were divided into the “with OAC” group and the “without OAC” group for further analysis.

### Outcomes and Follow-Up

The major adverse cardiac events (MACEs) for efficacy evaluation were defined as all-cause death, nonfatal myocardial infarction (MI), nonfatal stroke, and systemic embolism. The definitions for the outcomes were derived from the most recent updated guidelines (10). Safety outcomes of bleeding events were defined according to the Bleeding Academic Research Consortium (BARC) criteria of BARC ≥2 (25). Follow-up was performed via phone call or by a review of the medical records of readmission or repeat outpatient visits. All eligible patients were followed-up for at least 1 year or until the occurrence of MACEs. A well-thought-out follow-up questions by phone call were designed in advance with the certification of the Ethics Committee of Chinese PLA General Hospital. All the follow-ups by phone call were completed by the clinicians with qualifications in cardiology after receiving unified training. To verify the outcomes by phone call, the final judgement of all the endpoints was determined by the experts of the Endpoints Committee, the Clinical Drug Trial Center of Chinese PLA General Hospital.

### Statistical Analysis

The CHA_2_DS_2_-VASc score was used to assess the risk of ischemic events. The CHA_2_DS_2_-VASc score was calculated as congestive heart failure (1 point), hypertension (1 point), age ≥ 75 years (2 points), diabetes (1 point), stroke/transient ischemic attack/thromboembolism (2 points), vascular disease (prior myocardial infarction, peripheral artery disease, or aortic plaque: 1 point), age 65 to 74 years (1 point), and female sex (1 point) (26). A modified HAS-BLED score was used to assess the risk of bleeding. The modified HAS-BLED score was calculated as hypertension (1 point), abnormal renal and liver function (1 point each), stroke (1 point), bleeding (1 point), elderly (age > 65 years: 1 point), drugs or alcohol (1 point each) (27, 28). The labile international normalized ratio (INR) was not available in our study; hence, this factor was not included in the calculation of the modified HAS-BLED score. Continuous and categorical variables were described as the mean ± standard deviation (SD) or median (interquartile range [IQR]) and frequencies (percentages), respectively. Continuous variables were compared using *t*-tests, while categorical variables were compared using chi-square tests. To identify the independent predictive ability of OAC treatment on the efficacy and safety of adverse clinical outcomes, Cox multivariate models were used with the adjustment of the covariances, including the significantly different baseline clinical characteristics between the groups with or without OAC. Kaplan–Meier estimates of MACEs and bleeding events were used to construct time-to-event curves. All analyses were performed using SPSS (version 26.0), R software (version 3.6.0), and GraphPad Prism (version 8.0.1) software. All tests were two-tailed, and *P* values < 0.05 were considered statistically significant.

## Results

### Patient Characteristics

**Figure 1:**
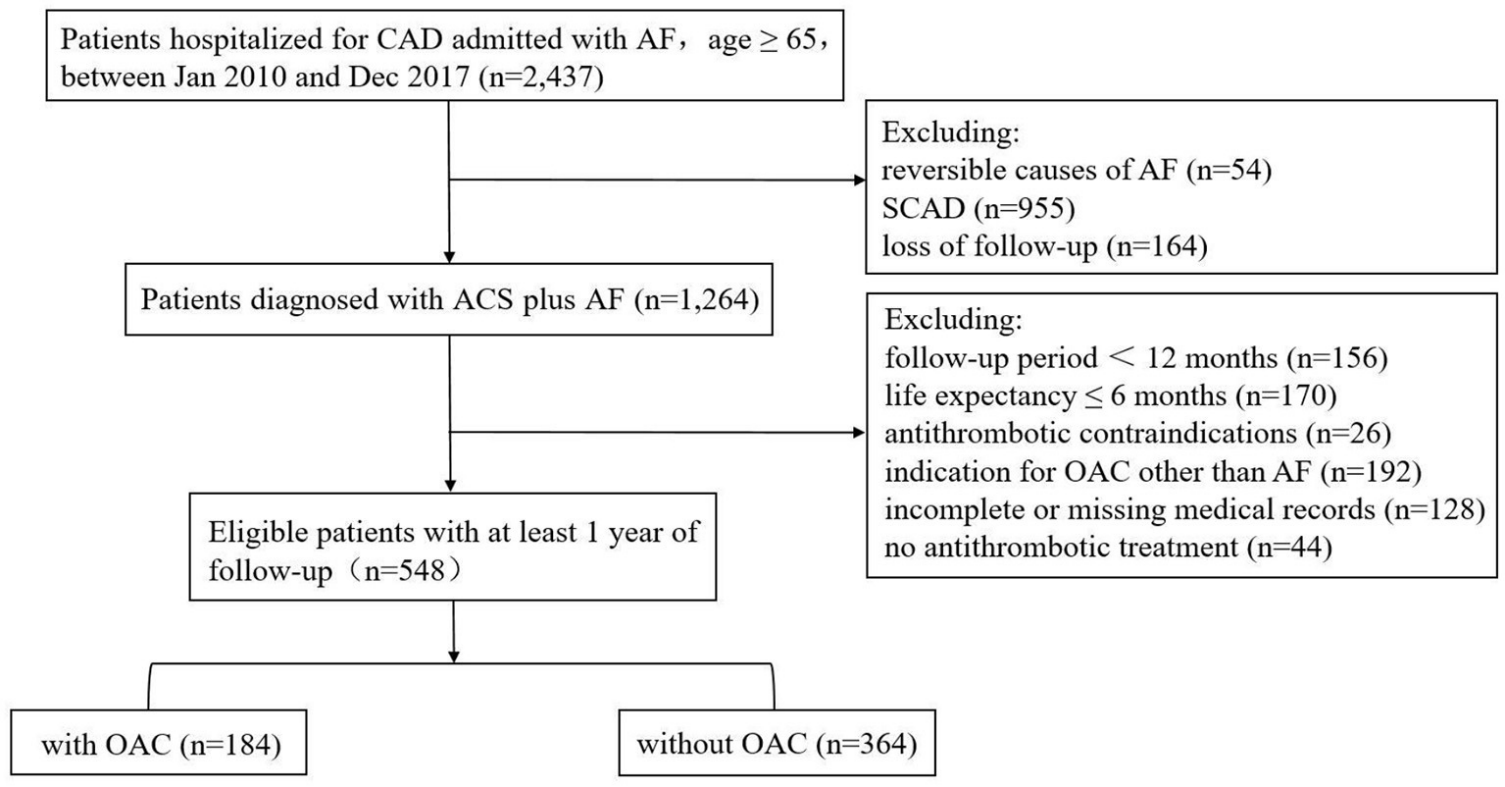
Flow chart over selection of the cohort. CAD, coronary artery disease; AF, atrial fibrillation; SCAD, stable coronary artery disease; OAC, oral anticoagulant.

**Figure 2:**
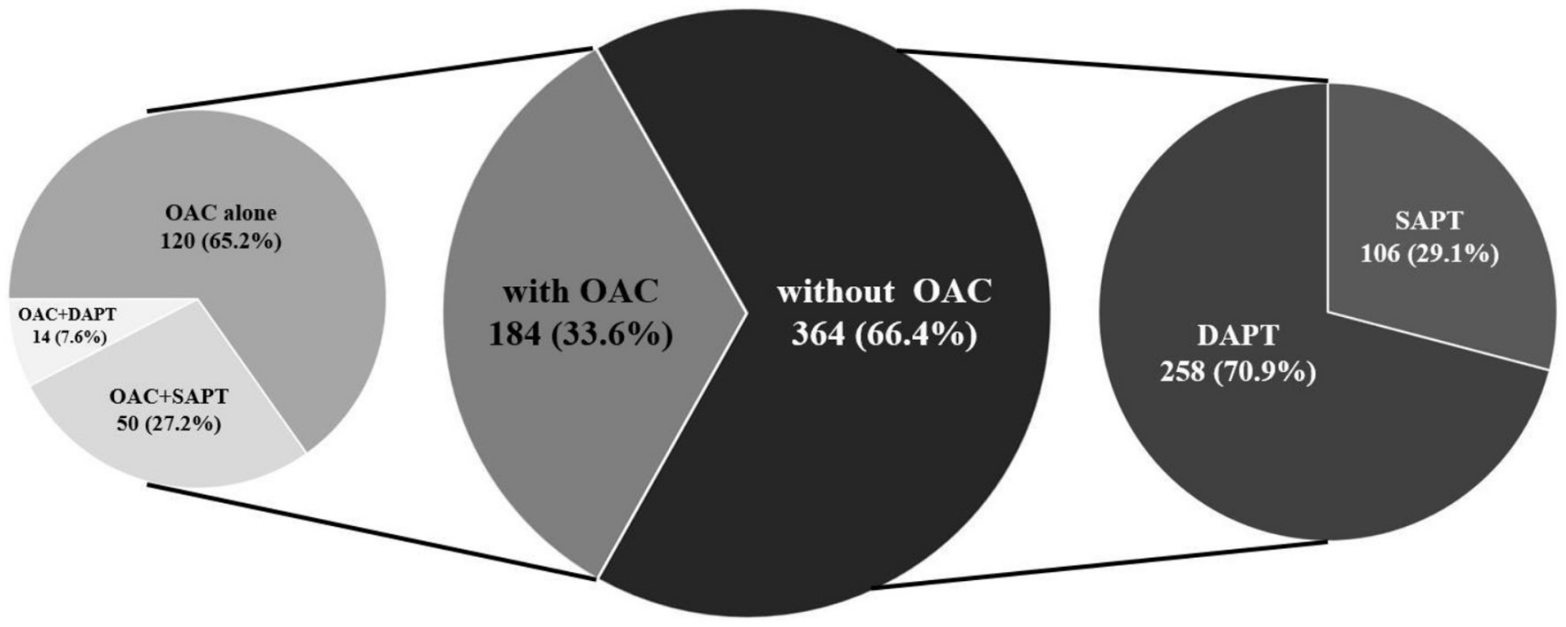
Prevalence of antithrombotic strategies. OAC, oral anticoagulant; DAPT, dual antiplatelet treatment; SAPT, single antiplatelet treatment.

**Figure 3:**
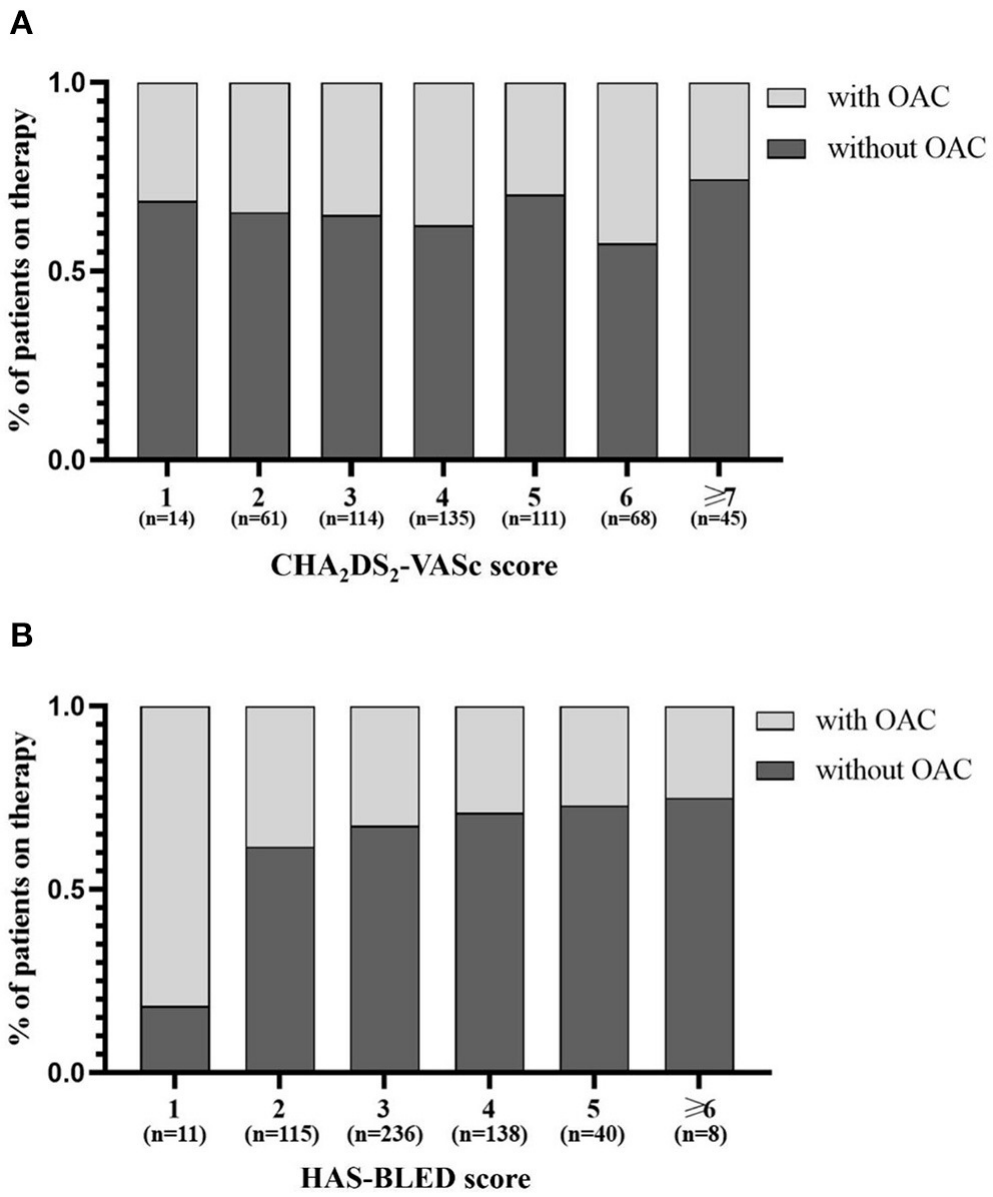
Distribution of antithrombotic strategies by CHA_2_DS_2_-VASc score **(A)** and HAS-BLED score **(B)**. OAC, oral anticoagulant.

Among the 2,437 continuously enrolled patients diagnosed with CAD and AF, the overall follow-up response rate of the cohort was 93.2%. A total of 548 eligible patients with ACS and AF were ultimately included in the analysis (Figure 1). The baseline characteristics of the patients according to the different treatment regimens with or without OAC are shown in Table 1. Overall, the patients had a mean age of 76 years (76 ± 6.6), and 251 (45.8%) were women. Among the included patients, 184 (33.6%) were treated with OACs, and 364 (66.4%) were not treated with OACs (Figure 2). All the included patients were followed up for 1 year, and a total of 290 patients were followed up for 5 years (with 83 patients treated with OAC and 207 without OAC). Compared to patients without OACs, patients treated with OACs were younger, had a higher prevalence of persistent AF and UA, and less often presented with paroxysmal AF, acute myocardial infarction (AMI), stent implantation and dual antiplatelet therapy (DAPT) (Table 1). With the increase in the CHA_2_DS_2_-VASc score, the percentage of patients treated with OACs did not increase accordingly (Figure 3A). With an increasing HAS-BLED score, the percentage of patients treated with OACs decreased accordingly (Figure 3B).

**Table 1:** Clinical baseline characteristics according to with or without OAC treatment.

**Characteristic**	**Total (*n* = 548)**	**With OAC (*n* = 184)**	**Without OAC (*n* = 364)**	***P* value**
Age (years, mean ± SD)	76 ± 6.6	75 ± 6.2	77 ± 6.7	**0.038**
Female (*n*, %)	251 (45.8)	92 (50.0)	159 (43.7)	0.161
BMI (kg/m^2^, mean ± SD)	24.9 ± 3.7	25.7 ± 3.5	24.5 ± 3.7	0.511
**Comorbidity (** ***n*** **, %)**				
Hypertension	414 (75.5)	144 (78.3)	270 (74.2)	0.293
Hyperlipidemia	145 (26.5)	50 (27.2)	95 (26.1)	0.788
Diabetes	187 (34.1)	65 (35.3)	122 (33.5)	0.673
HF	196 (35.8)	59 (32.1)	137 (37.6)	0.199
COPD	13 (2.4)	5 (2.7)	8 (2.2)	0.709
Renal Insufficiency	69 (12.6)	21 (11.4)	48 (13.2)	0.554
Chronic Renal Insufficiency	54 (9.9)	16 (8.7)	38 (10.4)	0.518
Malignant Tumor	56 (10.2)	14 (7.6)	42 (11.5)	0.149
**Type of AF (** ***n*** **, %)**				
Paroxysmal	291 (53.1)	82 (44.6)	209 (57.4)	**0.004**
Persistent	83 (15.1)	39 (21.2)	44 (12.1)	**0.005**
Unclassified	210	63	111	**-**
**History (** ***n*** **, %)**				
Previous MI	5 (0.9)	0 (0.0)	5 (1.4)	0.110
Previous stroke	74 (13.5)	28 (15.2)	46 (12.6)	0.404
Previous bleeding	28 (5.1)	11 (6.0)	17 (4.7)	0.511
**Clinical presentation (** ***n*** **, %)**				
UA	426 (77.7)	161 (87.5)	265 (72.8)	**<0.001**
AMI	87 (15.9)	16 (8.7)	71 (19.5)	**0.001**
**Concomitant medication (** ***n*** **, %)**				
Statins	513 (93.6)	170 (92.4)	343 (94.2)	0.406
β-blockers	451 (82.3)	158 (85.9)	293 (80.5)	0.119
ACEI/ARB	303 (55.3)	107 (58.2)	196 (53.8)	0.338
Diuretics	286 (52.2)	100 (54.3)	186 (51.1)	0.472
Calcium Antagonists	310 (56.6)	112 (60.9)	198 (54.4)	0.149
PPI	309 (56.4)	100 (54.3)	209 (57.4)	0.442
PCI	212 (38.7)	52 (28.3)	160 (44.0)	**0.001**
SAPT with aspirin	105 (19.2)	37 (20.1)	68 (18.7)	0.688
SAPT with clopidogrel	56 (10.2)	18 (9.8)	38 (10.4)	0.811
DAPT with aspirin and clopidogrel	287 (52.4)	58 (31.5)	229 (62.9)	**<0.001**
DAPT with aspirin and ticagrelor	34 (6.2)	5 (2.7)	29 (8.0)	**0.016**

*OAC, oral anticoagulant; SD, standard deviation; BMI, body mass index; HF, heart failure; COPD, chronic obstructive pulmonary disease; AF, atrial fibrillation; MI, myocardial infarction; UA, unstable angina; AMI, acute myocardial infarction; ACEI, angiotensin converting enzyme inhibitors; ARB, angiotensin receptor blocker; PPI, proton pump inhibitors; PCI, percutaneous coronary intervention; SAPT, single antiplatelet treatment; DAPT, dual antiplatelet treatment. The bold values indicates the P values < 0.05 that were considered statistically significant between the groups for comparison*.

### Efficacy and Safety of OAC Treatment in Elderly Patients With ACS and AF

**Figure 4:**
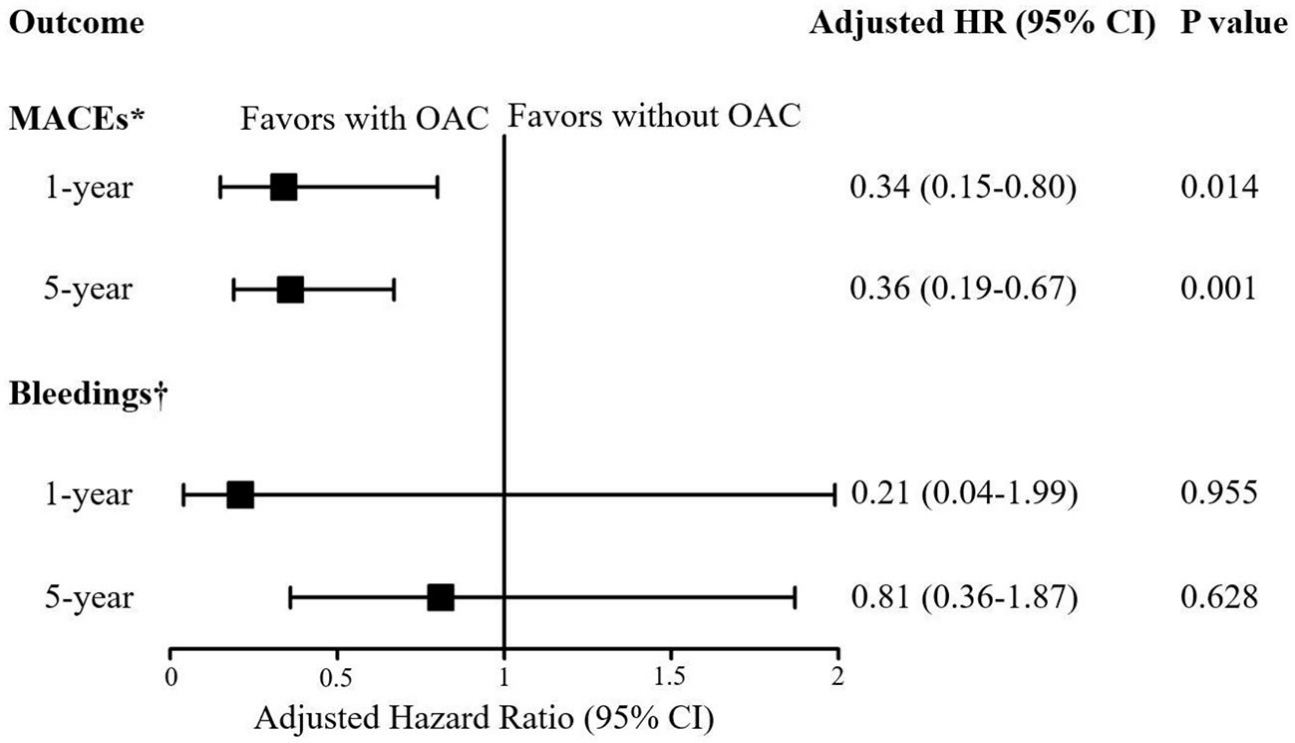
Forest plot for the risk of MACEs and bleedings in patients with or without OAC according to the length of follow-ups. *For MACEs, HR was adjusted by the variables including age, BMI, paroxysmal atrial fibrillation, persistent atrial fibrillation, unstable angina, acute myocardial infarction, renal insufficiency, heart failure, malignant tumor, percutaneous coronary intervention (PCI) and concomitant antiplatelet treatment;^†^For bleedings, HR was adjusted by age, BMI, paroxysmal atrial fibrillation, persistent atrial fibrillation, previous bleeding, unstable angina, acute myocardial infarction, renal insufficiency, heart failure, malignant tumor, PCI and concomitant antiplatelet treatment. HR, hazard ratio; CI, confidence interval; MACEs, Major Adverse Cardiovascular Events; Bleeding was defined as BARC ≥ 2; OAC, oral anticoagulant.

**Figure 5:**
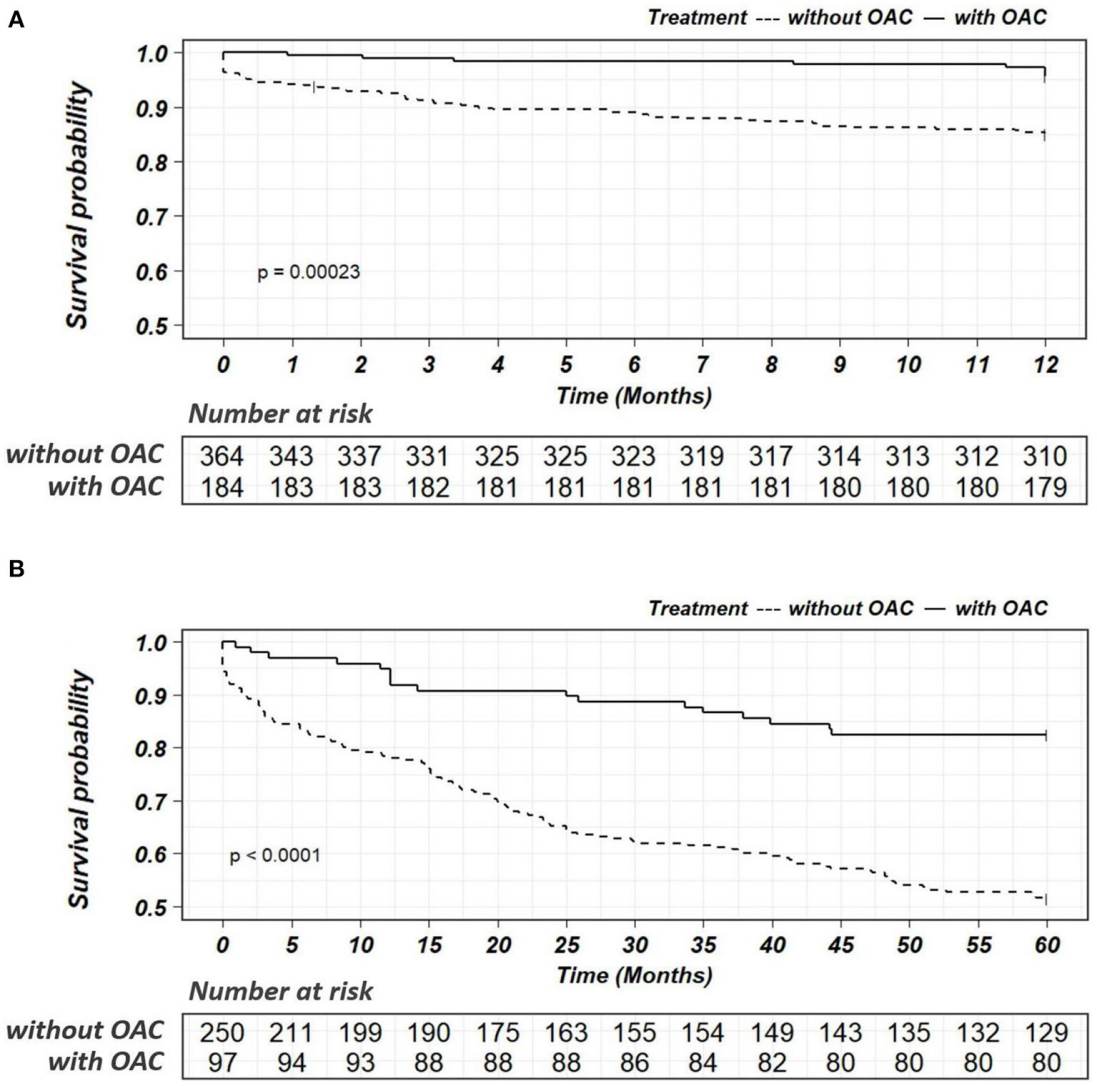
Kaplan-Meier survival curves for the endpoints of MACEs within a follow-up of 1 year **(A)** or 5 years **(B)** in patients with or without OAC. OAC, oral anticoagulant.

**Figure 6:**
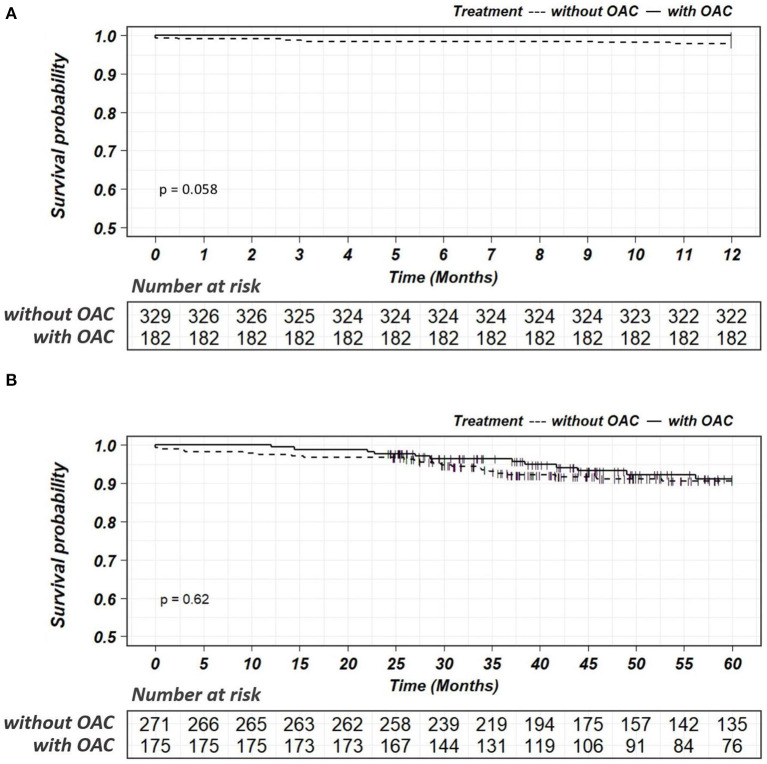
Kaplan-Meier survival curves for the endpoints of bleedings within a follow-up of 1 year **(A)** or 5 years **(B)** in patients with or without OAC. OAC, oral anticoagulant.

The median follow-up was 6.2 years (IQR: 4.2 to 8.5 years). During the follow-up, MACEs occurred in 153 (27.9%) patients, including all-cause death in 130, nonfatal MI in 4, nonfatal stroke in 27, and systemic embolism in 2 patients. After multivariable adjustment, the incidence of MACEs was significantly lower in patients treated with OACs than in those without OACs (9.2 vs. 37.4%, HR: 0.21, 95% CI: 0.10–0.41, *p* < 0.001) (Table 2). The efficacy of OACs could be found in patients followed-up for 1 year (4.3 vs. 15.1%, HR: 0.34, 95% CI: 0.15–0.80, *p* = 0.014) and 5 years (17.5 vs. 48.4%, HR: 0.36, 95% CI: 0.19–0.67, *p* = 0.001) (Figure 4). In terms of safety outcomes, bleeding events of BARC ≥ 2 occurred in 46 (8.4%) patients, including bleeding events of BARC ≥ 3 in 22 (4.0%) patients (Table 2). However, no significant difference in bleeding events was found between the patients with and without OAC (8.0 vs. 9.0%, HR: 1.17, 95% CI: 0.58–2.34, *p* = 0.667). Kaplan-Meier curves showed a significant decrease in MACEs (Figure 5) but not bleeding (Figure 6) among patients treated with OACs compared with patients without OACs at both the 1-year and 5-year follow-up.

**Table 2:** Risk of adverse clinical outcomes in patients with or without OAC.

**Outcomes**	**With OAC (No. of patients) (n=184)**	**Without OAC (No. of patients) (n=364)**	*Adjusted HR (95% CI)	***P* value**
MACEs	17 (9.2)	136 (37.4)	0.21 (0.10–0.41)	**<0.001**
All-cause death	10 (5.4)	120 (33.0)	0.10 (0.04–0.27)	**<0.001**
Cardiac death	5 (2.7)	35 (9.6)	0.32(0.11–0.94)	**0.039**
Fatal AMI	1 (0.5)	16 (4.4)	0.23(0.02–2.18)	0.198
Non-fatal MI	1 (0.5)	3 (0.8)	0.87 (0.06–11.91)	0.918
Non-fatal stroke	7 (3.8)	20 (5.5)	0.79 (0.28–2.23)	0.653
Systemic embolism	1 (0.5)	1 (0.3)	1.26 (0.04–41.08)	0.896
Bleedings	17 (8.0)	29 (9.0)	1.17 (0.58–2.34)	0.667
BARC ≥ 3	8 (4.3)	14 (3.8)	1.20 (0.44–3.26)	0.718
BARC ≥ 2	17 (8.0)	29 (9.0)	1.17 (0.58–2.34)	0.667

*For MACEs, HR was adjusted by the variables including age, BMI, paroxysmal atrial fibrillation, persistent atrial fibrillation, unstable angina, acute myocardial infarction, renal insufficiency, heart failure, malignant tumor, percutaneous coronary intervention (PCI) and concomitant antiplatelet treatment; For bleedings, HR was adjusted by age, BMI, paroxysmal atrial fibrillation, persistent atrial fibrillation, previous bleeding, unstable angina, acute myocardial infarction, renal insufficiency, heart failure, malignant tumor, PCI and concomitant antiplatelet treatment. OAC, oral anticoagulant; HR, hazard ratio; CI, confidence interval; MACEs, major adverse cardiovascular events; MI, myocardial infarction; AMI, acute myocardial infarction; BARC, Bleeding Academic Research Consortium criteria. The bold values indicates the P values < 0.05 that were considered statistically significant between the groups for comparison.

### Efficacy and Safety of NOACs vs. Warfarin Treatment in Elderly Patients With ACS and AF

We also performed a subgroup analysis of the efficacy and safety of NOAC treatment vs. warfarin treatment. A total of 94 (51.1%) patients were treated with NOACs, and 84 (45.6%) patients were treated with warfarin, excluding 6 (3.3%) patients who switched from warfarin to rivaroxaban during follow-up. The baseline characteristics according to the different treatment regimens (NOAC or warfarin) are shown in Supplementary Table. Multivariable Cox regression analysis showed that NOAC and warfarin were comparable in terms of efficacy (MACEs: 7.4 vs. 13.1%, HR: 0.50, 95% CI: 0.17–1.46, *p* = 0.204) and safety (bleeding: 6.4vs. 10.7%, HR: 0.36, 95% CI: 0.11–1.21, *p* = 0.099), respectively (Table 3). NOAC treatment yielded a marginally much reduction in all-cause mortality compared with warfarin treatment in elderly patients with ACS and AF (2.1 vs. 9.5%, adjusted HR: 0.18, 95% CI: 0.03–0.98, *p* = 0.047) (Table 3).

**Table 3:** Risk of adverse clinical outcomes in patients with warfarin or with NOAC.

**Outcomes**	**With NOAC (No. of patients) (*n* = 94)**	**With warfarin (No. of patients) (*n* = 84)**	*Adjusted HR (95% CI)	***P* value**
MACEs	7 (7.4)	11 (13.1)	0.50 (0.17–1.46)	0.204
All-cause death	2 (2.1)	8 (9.5)	0.18 (0.03–0.98)	**0.047**
Non-fatal MI	0 (0.0)	1 (1.2)	0.06 (0.02–8.17)	0.698
Non-fatal stroke	3 (3.2)	4 (4.8)	0.46 (0.08–2.50)	0.366
Systemic embolism	1 (1.0)	0 (0.0)	-	-
Bleedings	6 (6.4)	9 (10.7)	0.36 (0.11–1.21)	0.099
BARC ≥ 3	2 (2.1)	4 (4.8)	0.14 (0.02–1.10)	0.062
BARC ≥ 2	6 (6.4)	9 (10.7)	0.36 (0.11–1.21)	0.099

*For MACEs, HR was adjusted by the variables including age, paroxysmal atrial fibrillation, persistent atrial fibrillation, unstable angina, acute myocardial infarction, renal insufficiency, heart failure, percutaneous coronary intervention (PCI), β-blocker and concomitant antiplatelet treatment; For bleedings, HR was adjusted by age, paroxysmal atrial fibrillation, persistent atrial fibrillation, previous bleeding, unstable angina, acute myocardial infarction, renal insufficiency, heart failure, PCI, β-blocker and concomitant antiplatelet treatment. OAC, oral anticoagulant; HR, hazard ratio; CI, confidence interval; MACEs, major adverse cardiovascular events; MI, myocardial infarction; BARC, Bleeding Academic Research Consortium criteria. The bold values indicates the P values < 0.05 that were considered statistically significant between the groups for comparison.

## Discussion

In the present study, the major findings obtained from real-world elderly patients with comorbidities of ACS and AF are the following: (1) The selection of oral antithrombotic strategies is mainly affected by the risk of bleeding. (2) The application of OACs can reduce the risk of major ischemic events without increasing bleeding. This suggests that in real-world clinical practice, OACs could be safely applied in elderly patients with ACS and AF. To our knowledge, this is the first real-world study to investigate the efficacy and safety of OACs in elderly patients with both ACS and AF.

Therapy with OAC was recommended in patients with both ACS and AF after PCI or medical treatment, whichever initial plan was chosen (9). However, patients with both ACS and AF were less likely to receive appropriate antithrombotic therapy (23) and more likely to suffer from adverse outcomes (9, 22). Our study showed that only 33.6% of elderly patients with both ACS and AF were treated with OACs. The proportion was comparable to that recently reported (44.7%) in elderly Chinese patients with AF alone (21) and that reported (36.5%) in Chinese AF patients with a CHA_2_DS_2_-VASc score ≥2 (29). The reason for the underutilization of OACs in the present study could be attributed to the factors age, type of AF, AMI, PCI, and the concomitant use of DAPT. As we know, physicians in real-world clinical practice are reluctant to discontinue antiplatelet treatment, especially among PCI-treated ACS patients with AF, therefore discouraging physicians from prescribing OACs (30). In addition, elderly patients who presented with both ACS and AF were more likely to be prescribed DAPT rather than OAC by physicians (21, 31). The rate of the present elderly patients with ACS and AF underwent PCI was relatively low (38.7%), mostly attributing to the mild coronary stenosis (<50%), patients' unwillingness, contraindications for PCI, advanced age (>85), or recommending to cardiac surgery. Despite the lower percentage, there was absolutely an increased trend for PCI in the present elderly patients with ACS and AF with the increasing years (data not shown). However, even with the fewer patients underwent PCI, the OACs remained under-prescription. Moreover, the present study found that the ratio of OAC treatment showed a significant decrease with increasing HAS-BLED score but did not increase with increasing CHA_2_DS_2_-VASc score. This result indicated that the antithrombotic strategies in real-world elderly patients with both ACS and AF were affected mostly by the risk of bleeding instead of ischemic events. However, the application of OACs in the present elderly patients with ACS and AF conferred a 4 times lower risk of death, without an increase in bleeding. Similar efficacy of OACs was found in elderly individuals with AF (21, 30, 32). Therefore, greater efforts to improve the prescription of OACs in the elderly are necessary, especially among older subjects with ACS and AF. It was recommended by consensus that a short course of dual therapy with OAC and an antiplatelet agent (preferably P2Y_12_) should be considered as a preferred antithrombotic strategy in the therapeutic process of patients with both ACS and AF (9, 33), and OAC should be applied in the long term of the patients' antithrombotic procedure (9). In our study, we found that even after a long-term follow-up of 5 years, OACs could still safely reduce mortality in elderly patients with both ACS and AF.

The comparative efficacy and safety of NOAC and warfarin treatment among patients with AF has been the focus of clinical trials. The Asian subgroup analysis (34) of four major randomized control trials showed that NOACs were comparable to warfarin in the reduction in ischemic stroke and myocardial infarction. However, all-cause mortality and major bleeding were significantly lower in patients treated with NOACs. Consistent with the results, although the sample size was small, our study in elderly patients with ACS and AF validated the advantage of NOAC over warfarin for the reduction in all-cause mortality and major bleeding. This result indicated that NOACs could be safely applied in elderly patients with ACS and AF, especially in those who are at higher risk of bleeding when treated via comedication with antiplatelet agents.

### Limitations

This study has several limitations, which should be mentioned. First, due to the relatively small sample size of the whole cohort, it is difficult to compare the outcomes among subgroups with various combinations of antiplatelet and anticoagulation strategies. However, we compared the efficacy and safety between NOAC (*n* = 94) and warfarin (*n* = 84) in elderly patients with ACS and AF. The advantage of NOAC over warfarin could be observed in the form of the lower risk of all-cause mortality and major bleeding (BARC ≥ 3), with marginal significance. These results are in agreement with previous studies, indicating the benefit of NOAC compared with warfarin extending to older adults with AF (35–37). Second, the consecutively recruited patients were followed-up for various lengths of time, ranging from 1 to 9 years. However, the efficacy and safety outcomes were compared in patients with the same length of follow-up, including 1-year and 5-year intervals. During both follow-up intervals, treatment with OAC was consistently associated with a lower risk of MACEs and bleeding. Finally, the present observational study was retrospectively designed and based on a single center cohort. The findings should be further validated in multicenter prospectively recruited patients.

## Conclusion

Antithrombotic therapy with OACs in elderly patients with ACS and AF could decrease the risk of ischemic events without increasing bleeding. In real-world practice, it is necessary to strengthen the awareness of anticoagulant treatment in elderly patients with ACS and AF.

## Data Availability Statement

The original contributions presented in the study are included in the article/Supplementary Material, further inquiries can be directed to the corresponding author.

## Ethics Statement

The studies involving human participants were reviewed and approved by Ethics Committee of Chinese PLA General Hospital. The patients/participants provided their written informed consent to participate in this study.

## Author Contributions

TY: study concept and design and critical revision of the manuscript for important intellectual content. YWu, HL, LQ, YWa, SZ, YZ, and ZW: acquisition of data. YWu, HL, LQ, and TY: analysis and interpretation of data. YWu, HL, and TY: drafting of the manuscript. All authors contributed to the article and approved the submitted version.

## Funding

This work was supported by grants from the National Natural Science Foundation of China (Nos. 81870262 and 82170352) and Special Project on Logistics Health Care (No. 22BJZ31).

## Conflict of Interest

The authors declare that the research was conducted in the absence of any commercial or financial relationships that could be construed as a potential conflict of interest.

## Publisher's Note

All claims expressed in this article are solely those of the authors and do not necessarily represent those of their affiliated organizations, or those of the publisher, the editors and the reviewers. Any product that may be evaluated in this article, or claim that may be made by its manufacturer, is not guaranteed or endorsed by the publisher.
