# Supplementary material for: Efficacy and Safety of Antithrombotic Therapy With Oral Anticoagulants in Real-World Elderly Patients With Acute Coronary Syndrome and Atrial Fibrillation
Source: Front Cardiovasc Med. 2022 Jun 29;9:923684. doi: 10.3389/fcvm.2022.923684 (PMC9276998; doi:10.3389/fcvm.2022.923684)
Supplement: Supplementary file 1 [file Table_1.docx]

**Supplement Table Baseline characteristics of patients with OAC.**

| **Characteristic** | **Total**  **(n=178)** | **with NOAC**  **(n=94)** | **with VKA**  **(n=84)** | **P value** |
| --- | --- | --- | --- | --- |
| Age (years, mean ± SD) | 75±6.2 | 77±6.7 | 73±5.0 | **<0.001** |
| Female (n, %) | 92 (51.7) | 45 (47.9) | 47 (56.0) | 0.354 |
| BMI (kg/m^2^, mean ± SD) | 25.7±3.5 | 25.9±3.4 | 25.4±3.5 | 0.340 |
| **Comorbidity (n, %)** |  |  |  |  |
| Hypertension | 140 (78.7) | 76 (80.9) | 64 (76.2) | 0.566 |
| Hyperlipidemia | 48 (27.0) | 26 (27.7) | 22 (26.2) | 0.959 |
| Diabetes | 64 (36.0) | 33 (35.1) | 31 (36.9) | 0.926 |
| HF | 58 (32.6) | 22 (23.4) | 36 (42.9) | **0.009** |
| COPD | 4 (2.2) | 2 (2.1) | 2 (2.4) | 1.000 |
| Renal Insufficiency | 19 (10.7) | 9 (9.6) | 10 (11.9) | 0.795 |
| Chronic Renal Insufficiency | 14 (7.9) | 7 (7.4) | 7 (8.3) | 1.000 |
| Malignant Tumor | 14 (7.9) | 7 (7.4) | 7 (8.3) | 1.000 |
| **Type of AF (n, %)** |  |  |  |  |
| Paroxysmal | 81 (45.5) | 49 (52.1) | 32 (38.1) | 0.084 |
| Persistent  Unclassified | 37 (20.8)  60 | 15 (16.0)  30 | 22 (26.2)  30 | 0.135  - |
| **History (n, %)**  Previous MI | 178 (100.0) | 94 (100.0) | 84 (100.0) | - |
| Previous stroke | 27 (15.2) | 15 (16.0) | 12 (14.3) | 0.919 |
| Previous bleeding | 10 (5.6) | 6 (6.4) | 4 (4.8) | 0.886 |
| **Clinical presentation (n, %)** |  |  |  |  |
| UA | 155 (87.1) | 80 (85.1) | 75 (89.3) | 0.544 |
| AMI | 16 (9.0) | 11 (11.7) | 5 (6.0) | 0.282 |
| **Concomitant medication (n, %)** |  |  |  |  |
| Statins | 164 (92.1) | 87 (92.6) | 77 (91.7) | 1.000 |
| β-blockers | 152 (85.4) | 75 (79.8) | 77 (91.7) | **0.043** |
| ACEI/ARB | 105 (59.0) | 57 (60.6) | 48 (57.1) | 0.748 |
| Diuretics | 97 (54.5) | 51 (54.3) | 46 (54.8) | 1.000 |
| Calcium Antagonists | 107 (0.1) | 57 (60.6) | 50 (59.5) | 1.000 |
| PPI | 88 (49.4) | 49 (52.1) | 39 (46.4) | 0.542 |
| PCI | 50 (28.1) | 30 (31.9) | 20 (23.8) | 0.301 |
| SAPT with aspirin | 37 (20.8) | 18 (19.1) | 19 (22.6) | 0.701 |
| SAPT with clopidogrel | 17 (9.6) | 9 (9.6) | 8 (9.5) | 1.000 |
| DAPT with aspirin and clopidogrel | 55 (30.9) | 35 (37.2) | 20 (23.8) | 0.076 |
| DAPT with aspirin and ticagrelor | 5 (2.8) | 3 (3.2) | 2 (2.4) | 1.000 |

OAC: oral anticoagulant; SD: standard deviation; BMI: body mass index; HF: heart failure; COPD: chronic obstructive pulmonary disease; AF: atrial fibrillation; MI: myocardial infarction; UA: unstable angina; AMI: acute myocardial infarction; ACEI: angiotensin converting enzyme inhibitors; ARB: angiotensin receptor blocker; PPI: proton pump inhibitors; PCI: percutaneous coronary intervention; SAPT: single antiplatelet treatment; DAPT: dual antiplatelet treatment.
